# Computed Tomography Diagnostic Reference Levels for Routine Pediatric Examinations in Saudi Arabia: A Systematic Review

**DOI:** 10.7759/cureus.71072

**Published:** 2024-10-08

**Authors:** Ahmad Abanomy

**Affiliations:** 1 Radiological Science Department, College of Applied Medical Science, King Saud University, Riyadh, SAU

**Keywords:** computed tomography, diagnostic reference levels, dose length product, pediatric ct, volumetric ct dose index

## Abstract

This work aims to review the diagnostic reference levels reported for pediatric patients who underwent routine CT exams in Saudi Arabia. The literature search was conducted in prominent search engines, including PubMed, Science Direct, Google Scholar, EBSCO, ProQuest, and Saudi Digital Library (SDL), which serve as important sources in the fields of radiology, radiography, and medical physics. The search was conducted to identify previous studies that have reported diagnostic reference levels (DRLs) for pediatric CT examinations. Only studies that specifically assessed DRLs in pediatric patients were considered for inclusion. On careful evaluation, eight publications were ultimately chosen out of the 31 that were originally considered. An assessment was made on the volume computed tomographic dose index (CTDIvol) and the dose-length product (DLP) values. The reported range of mean values for DLP (mGy.cm) included in this review for brain, chest, abdomen, abdomen and pelvis, and CAP (chest, abdomen, and pelvis) was 172-1881, 27.3-458.9, 94.5-790.1, 78-236, and 94.2, respectively. Moreover, the reported range of mean values for CTDIvol (mGy) included in this review for the brain, chest, abdomen, abdomen and pelvis, and CAP were 11.7-79.9, 1.2-20.1, 7.4-22.6, 2.6-5.5, and 3.6, respectively. The review emphasizes the need for standardized imaging protocols and DRLs in pediatric CT examinations to optimize radiation doses and patient safety. It recommends the development of annually reviewed comprehensive national DRLs in Saudi Arabia and improved training for technologists and physicians involved in pediatric CT imaging.

## Introduction and background

Computed tomography (CT) plays a crucial role as a diagnostic imaging technique. By generating high-resolution, cross-sectional visualizations of internal body structures, CT scans enable medical professionals to identify and assess various ranges of health conditions [1]. However, the use of ionizing radiation in CT scans requires careful management to minimize potential risks, especially in vulnerable populations such as pediatric patients. Children exhibit a higher tendency to develop radiation-induced cancer compared to adults due to several factors. Primarily, their tissues are in a phase of rapid growth, and their cells divide more frequently, rendering them more vulnerable to the mutagenic effects of ionizing radiation [1]. To ensure that the ionizing radiation dose remains within a "safe" threshold, a strategy was implemented to emphasize the necessity of optimizing radiation doses for patients, particularly pediatric patients. The International Commission on Radiological Protection (ICRP) 135 introduced diagnostic reference levels (DRLs) in its publication [2]. Prior to this, the concept of establishing an exploratory level for standard radiographic procedures had already been suggested. According to the Commission, a DRL is an investigation level employed to enhance protection during medical exposure to patients in diagnostic and interventional procedures [3]. DRLs have been established as a key strategy in optimizing radiation doses in medical imaging, which refer to the radiation dosage levels used for common medical tests on groups of average-sized people or standard phantoms, using widely categorized types of equipment. They serve as benchmarks for medical practitioners to compare their practices against standard levels, ensuring that doses are kept as low as reasonably achievable (ALARA) while maintaining diagnostic image quality [4]. Establishing and adhering to pediatric-specific DRLs is crucial to safeguard this vulnerable population. Pediatric DRLs help in standardizing practices, reducing unnecessary radiation exposure, and promoting the use of optimized imaging protocols tailored to children's unique anatomical and physiological characteristics [5].

The two primary units of radiation dose for CT are the volume computed tomographic dose index (CTDIvol), measured in mGy, and the dose-length product (DLP), measured in mGy.cm. Essentially, CTDIvol represents the average dose absorbed by a specific phantom at a given scanner output (i.e., a set of scanning parameters or "protocol"). In contrast, the DLP is adjusted for the scan length, providing an estimate of the total absorbed dose [6].

In Saudi Arabia, the increasing utilization of CT imaging in pediatric patients aligns with global trends in advanced diagnostic technologies. However, the establishment and implementation of pediatric DRLs in the region are still developing [7]. A study in the United Arab Emirates emphasized the necessity of regularly reviewing DRLs to optimize radiation doses for pediatric head CT scans, proposing specific values per age group to ensure patient safety and protocol standardization [8]. Furthermore, the need for region-specific DRLs is crucial to adapt to local practices and patient demographics, considering variations in equipment, protocols, and populations across different regions [9,10]. This underscores the importance of setting and adhering to tailored DRLs to minimize radiation risks and enhance the quality of pediatric CT imaging in the region. This paper aims to review the diagnostic reference levels reported for pediatric patients who underwent routine CT exams in Saudi Arabia.

## Review

Methodology

Design and Search Strategy

**Figure 1:**
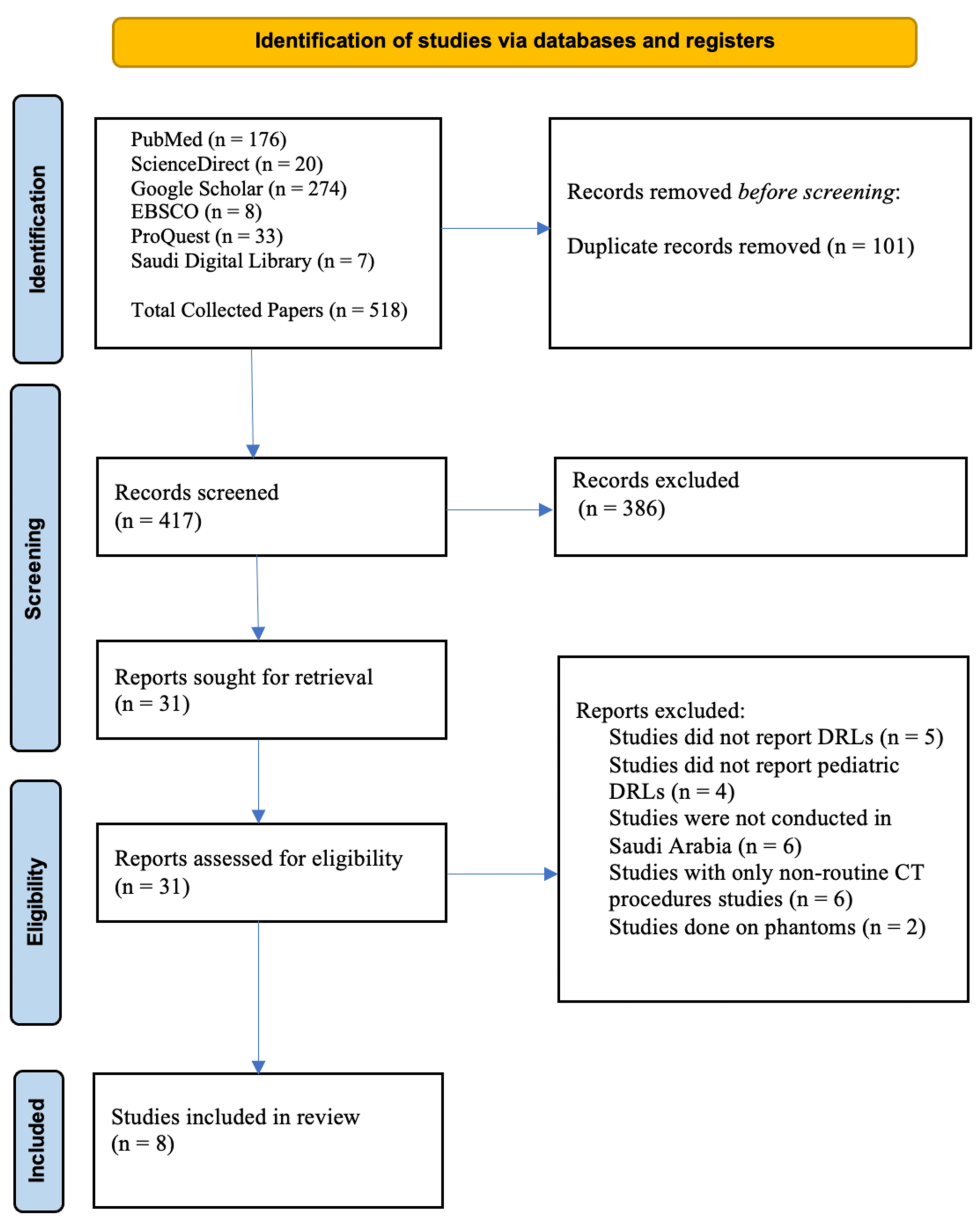
PRISMA flow chart showing how the articles were identified Source: [12] PRISMA: Preferred Reporting Items for Systematic Reviews

The Preferred Reporting Items for Systematic Reviews (PRISMA) flow chart was used as a search strategy, as depicted in Figure 1 [11]. The PRISMA flow chart serves as an effective tool for documenting the article selection process at each screening phase. The databases chosen for the literature search included PubMed, Science Direct, Google Scholar, EBSCO, ProQuest, and Saudi Digital Library (SDL), which are prominent sources in the fields of radiology, radiography, and medical physics. Articles published before 1990 were excluded from the review as the concept of DRLs had not been established at that time [12], and it was noted that no articles relevant to the study were published before 2010, thereby omitting outdated historical dose data from older scanners. The search term for the identification of articles is shown in Table 1. Inclusion criteria for the studies include those reporting diagnostic reference levels (DRLs) for pediatric patients and those conducted within Saudi Arabian healthcare facilities. Exclusion criteria encompass studies on adult populations, studies that do not specify the CT examination protocols, reviews, editorials, and non-peer-reviewed articles.

**Table 1:** The terms used to identify the relevant articles DRL: diagnostic reference level; DLP: dose-length product

Intervention	Cohort
Diagnostic Reference Levels	Pediatric CT
DRLs	Pediatric CT Exams
Dose Reference Levels	CT Saudi Arabia
DLP	Pediatric CT Saudi Arabia

Risk of bias

Using the ROBINS-I (risk of bias in non-randomized research - of interventions) tool [13], we assessed the potential for bias in all the included studies. Seven types of bias are examined by this tool: confounding, participant selection, intervention classification, missing data, outcome measurement, result selection, and variations from intended interventions. Each domain was evaluated as possessing low, moderate, serious, or critical risk of bias. This thorough evaluation is essential in systematic reviews as it aids in identifying potential bias elements that may compromise the validity and reliability of the study findings. The "Robvis" tool was used to create traffic light graphs following Cochrane guidelines [14].

Results

The systematic literature search resulted in 518 articles, which were assessed using the Rayyan AI tool for systematic reviews [15]. Subsequently, 101 duplicate articles were eliminated from further consideration. An additional 387 articles were excluded due to their studies not being conducted within health institutes in Saudi Arabia. Thirty-one studies were reviewed, and 23 papers were excluded based on specific criteria, including studies conducted between 2000 and 2024 involving pediatric CT scans in Saudi Arabia. Ultimately, eight articles met the inclusion criteria and were incorporated into the systematic review, as depicted in Figure 1.

**Figure 2:**
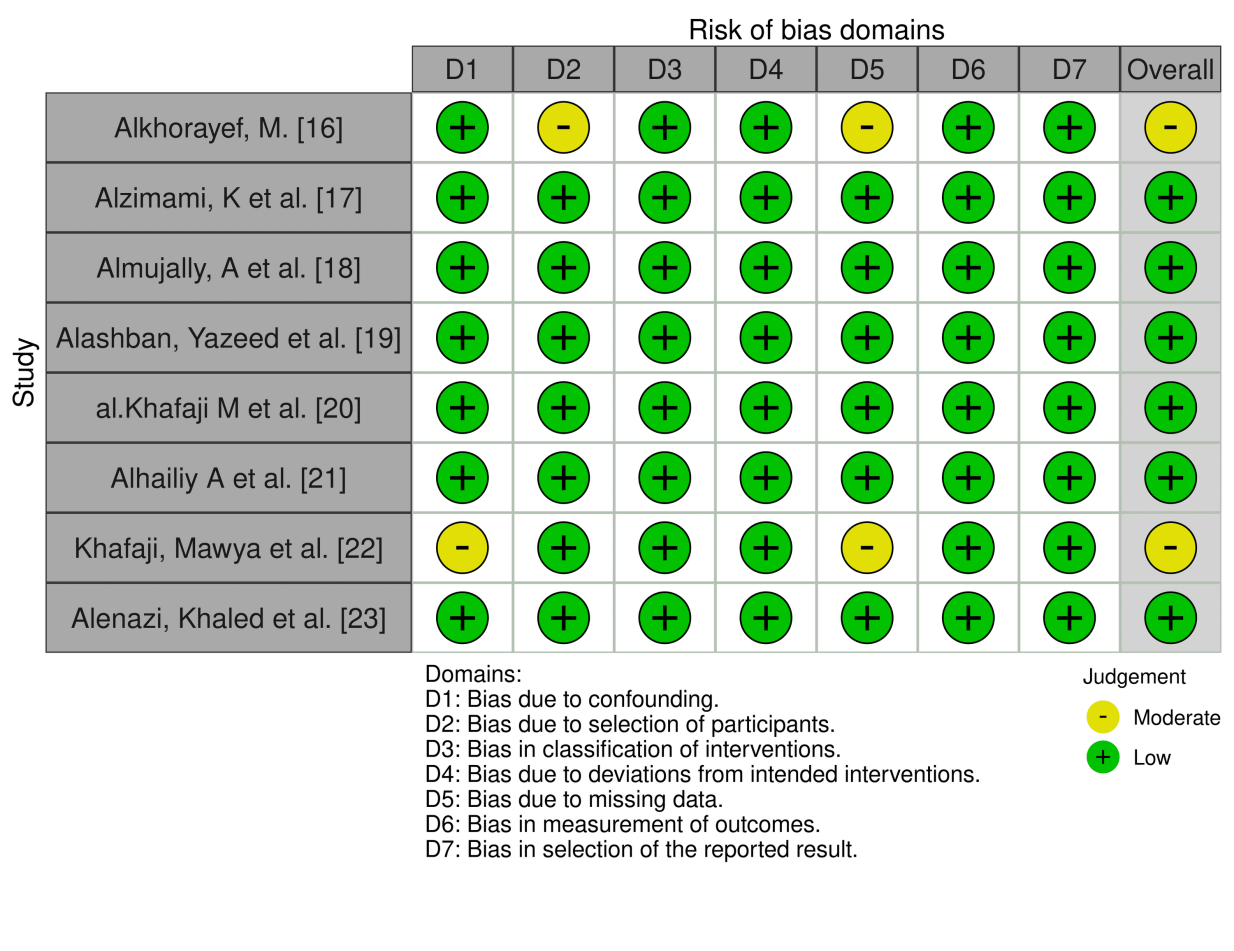
Risk of bias plot using the ‘Robvis’ tool developed by Cochrane Source: [14]

The studies included in this review are presented in Table 2. The summary of DLP and CTDIvol for routine CT examinations of the selected studies is shown in Table 3. Also, an assessment of bias risk was conducted using the ROBINS-I tool, following Cochrane guidelines (Figure 2) [13,14].

**Table 2:** Summary of characteristics of selected studies CTDIvol: volume computed tomographic dose index; DLP: dose-length product

Author/s	Year	Country	Method	Sample Size	No. of CT Machines	No. of Hospitals	Study Approach	Part Studied	DRLs
Alkhorayef, M. [16]	2020	Saudi Arabia	Retrospective	59	3	3	Age; Weight; Height	Brain, Chest, Abdomen	DLP
Alzimami, K et al. [17]	2021	Saudi Arabia	Retrospective	83	1	1	Age; Weight; Height; BMI	Chest, Abdomen	CTDIvol DLP
Almujally, A et al. [18]	2022	Saudi Arabia	Retrospective	48	4	4	CT machine	Brain, Abdomen	CTDIvol DLP
Alashban, Yazeed et al. [19]	2022	Saudi Arabia	Retrospective	76	3	3	Age-based	Brain, Chest, Abdomen	CTDIvol DLP
Al Khafaji M et al. [20]	2023	Saudi Arabia	Retrospective	1005	1	1	Age-based	Head, Chest	CTDIvol DLP
Alhailiy A et al. [21]	2023	Saudi Arabia	Retrospective	226	3	3	Age-based	Brain	CTDIvol DLP
Khafaji, Mawya et al. [22]	2024	Saudi Arabia	Retrospective	3714	--	1	Age-based	Brain Chest Abdomen &Pelvis	CTDIvol DLP
Alenazi, Khaled et al. [23]	2024	Saudi Arabia	Retrospective	255	3	2	Age-based	Abdomen &Pelvis Chest CAP	CTDIvol DLP

**Table 3:** Summary of DRLs for routine CT exams of selected studies *: For each hospital; §: Before COVID19 and after COVID19; $: For each age group CAP: chest, abdomen, and pelvis; CTDIvol: volume computed tomographic dose index; DRL: diagnostic reference level

Exam	Alkhorayef, M. [16]		Alzimami, K et al. [17]		Almujally, A et al. [18]		Alashban, Yazeed et al. [19]		al. Khafaji M et al. [20]		Alhailiy A et al. [21]		Khafaji, Mawya et al. [22]		Alenazi, Khaled et al. [23]		EDRL [24]	
	CTDIvol, mGy	DLP, mGy cm	CTDIvol, mGy	DLP, mGy cm	CTDIvol, mGy	DLP, mGy cm	CTDIvol, mGy	DLP, mGy cm	CTDIvol, mGy	DLP, mGy cm	CTDIvol, mGy	DLP, mGy cm	CTDIvol, mGy	DLP, mGy cm	CTDIvol, mGy	DLP, mGy cm	CTDIvol, mGy	DLP, mGy cm
Brain	--	300^*^, 328.8^*^, 350^*^	--	--	34.9^*^, N/A^*^, 53.9^*^, 27^*^	569.8^*^, 1881^*^, 482^*^, 375^*^	79.92	669.26	15.54^§^, 19.29^§^	775.4^§^, 1151.9^§^	20.9^$^, 27.6^$^, 30.5^$^, 32.3^$^	312.6^$^, 470.3^$^, 519.5^$^, 574^$^	12.03^*^, 11.72^*^, 14.48^*^, 18.34^*^	172.13^*^, 176.52^*^, 242.95^*^, 320.34^*^	--	--	24^$^, 28^$^, 40^$^, 50^$^	300^$^, 385^$^, 505^$^, 650^$^
Chest	--	62.5^*^, 73.1^*^, 80.4^*^	2	42	--	--	20.10	458.90	3.11^§^, 4.31^§^	224.9^§^, 94.5^§^	--	--	1.2	27.38	5.5	131.5	1.4^$^, 1.8^$^, 2.7^$^, 3.7^$^, 5.4^$^	35^$^, 50^$^, 70^$^, 115^$^, 200^$^
Abdomen	--	340.8^*^, 94.5^*^, 295.1^*^	--	--	17.4^*^, N/A^*^, 47^*^, 7.4^*^	790.1^*^, 505^*^, 976^*^, 336^*^	22.68	691.77	--	--	--	--	--	--	--	--	3.5^$^, 5.4^$^, 7.3^$^, 13^$^	45^$^, 120^$^, 150^$^, 210^$^, 480^$^
Abdomen & Pelvis	--	--	3.8	116	--	--	--	--	--	--	--	--	5.29^*^, 2.62^*^, 3.9^*^	132.54^*^, 78.03^*^, 106.62^*^	5.5	236	--	--
CAP	--	--	--	--	--	--	--	--	--	--	--	--	--	--	3.6	94.2	--	--

Discussion

Over the past 20 years, the application of CT in pediatric healthcare has markedly increased, consequently elevating the risk of radiation-induced cancers, particularly in abdominal and pelvic CT examinations. In response, CT manufacturers, hospitals, and imaging centers have implemented various strategies to optimize radiation doses for pediatric patients. Methods to decrease radiation exposure in medical procedures involve the implementation of incremental reconstruction techniques, regulation of tube voltage and current, and using bow tie filters. Additionally, protocol optimization has been achieved by establishing protocols based on age, weight, and specific clinical indications to enhance dose reduction [25,26].

The weight bands proposed by ICRP 135 and the diagnostic reference levels for pediatric imaging (PiDRL) project in Europe [24] are as follows: less than 5 kg, 5-15 kg, 15-30 kg, 30-50 kg, and 50-80 kg. The suggested age groups are <1 month, <1-4 years, <10-14 years, and <14-18 years. The PiDRL project and ICRP 135 suggest future research to determine pediatric DRLs using age and weight ranges [3,24]. Different methodologies for calculating and reporting dose metrics (e.g., CTDIvol, DLP) observed from the included papers could lead to inconsistencies (Table 3).

Six studies reported dose metrics for brain and chest procedures. Three studies reported dose metrics for abdomen and AP procedures. Only one study reported dose metrics for CAP. The reported range of mean values for DLP (mGy.cm) included in this review for brain, chest, abdomen, AP, and CAP was 172-1881, 27.3-458.9, 94.5-790.1, 78-236, and 94.2, respectively. Moreover, the reported range of mean values for CTDIvol (mGy) included in this review for brain, chest, abdomen, AP, and CAP were 11.7-79.9, 1.2-20.1, 7.4-22.6, 2.6-5.5, and 3.6, respectively.

In 2022, the Saudi Food and Drug Authority (SFDA) published a National Diagnostic Reference Levels (NDRLs) report [27]; however, they only reported NDRLs for pediatric head CT examinations. These values were reported for two age group categories as follows: for children aged 0-5 years, the DLP was 482 mGy-cm, and for those aged 6-15 years, it was 697 mGy-cm. And the CTDIvol was 28 mGy for children aged 0-5 years and 42 mGy for those aged 6-15 years. From the studies that reported mean values of DLP for head CT examination, Alashban Yazeed et al. [19] and Al Khafaji M et al. [22] exceeded the values reported by SFDA’s NDRLs. Moreover, Alhailiy A et al. exceeded the values reported by European Commission DRL guidelines (Table 3) [21,24]. The quality of all included studies was assessed as moderate to high, with a generally low risk of bias according to the ROBINS-I risk of bias tool, indicating a reasonable level of validity within the research.

Since the SFDA only released the pediatric NDRLs for one CT scan, it is advised that additional pediatric NDRLs for routine CT exams, including the chest, abdomen, abdomen pelvis, and CAP scans, also be reported for future reference. It is also advised that NDRLs be reported on an annual basis. Moreover, it is recommended to utilize the European Commission’s grouping categories [24] for future DRL research to enhance the reporting system and facilitate result comparison. Finally, the recommendations from ICRP 135 to create DRLs using weight, equivalent diameter, or cross-sectional area must be considered for future work in establishing NDRLs [3].

## Conclusions

This review highlights the critical need for standardized imaging protocols and establishing DRLs to optimize radiation doses and ensure patient safety in pediatric CT examinations. The wide range of reported mean values for DLP and CTDIvol across different CT examinations emphasizes the importance of dose optimization and standardization of imaging practices to minimize radiogenic risks for pediatric patients. It also recommends the development of comprehensive national DRLs in Saudi Arabia, as well as improved training and skills of technologists and physicians involved in pediatric CT imaging, to ensure patient safety and optimize radiation doses. Future research should also consider the ICRP 135 recommendation to create DRLs using weight, equivalent diameter, or cross-sectional area to further enhance dose optimization.
